# Contrasting population differentiation in two sympatric *Triplophysa* loaches on the Qinghai–Tibet Plateau

**DOI:** 10.3389/fgene.2022.958076

**Published:** 2022-08-25

**Authors:** Ling Jin, Zitong Li, Chongnv Wang, Yingnan Wang, Xinxin Li, Jian Yang, Yahui Zhao, Baocheng Guo

**Affiliations:** ^1^ Key Laboratory of Zoological Systematics and Evolution, Institute of Zoology, Chinese Academy of Sciences, Beijing, China; ^2^ University of Chinese Academy of Sciences, Beijing, China; ^3^ CSIRO Agriculture and Food, Canberra, ACT, Australia; ^4^ Assessment and Resource Conservation in Middle and Lower Reaches of the Yangtze River, Freshwater Fisheries Research Center, Chinese Academy of Fishery Sciences, Wuxi, China; ^5^ Center for Excellence in Animal Evolution and Genetics, Chinese Academy of Sciences, Kunming, China; ^6^ Academy of Plateau Science and Sustainability, Qinghai Normal University, Xining, China

**Keywords:** population genomics, transcriptomics, otolith EPMA, linkage disequilibrium, salinity

## Abstract

Genetic differentiation in aquatic organisms is usually shaped by drainage connectivity. Sympatric aquatic species are thus expected to show similar population differentiation patterns and similar genetic responses to their habitats. Water bodies on the Qinghai–Tibet Plateau (QTP) have recently experienced dramatic physicochemical changes, threatening the biodiversity of aquatic organisms on the “roof of the world.” To uncover ecological genetics in Tibetan loaches (*Triplophysa*)—the largest component of the QTP ichthyofauna—we characterized population differentiation patterns and adaptive mechanisms to salinity change in two sympatric and phylogenetically closely related Tibetan loaches, *T. stewarti* and *T. stenura*, by integrating population genomic, transcriptomic, and electron probe microanalysis approaches. Based on millions of genome-wide SNPs, the two Tibetan loach species show contrasting population differentiation patterns, with highly geographically structured and clear genetic differentiation among *T. stewarti* populations, whereas there is no such observation in *T. stenura*, which is also supported by otolith microchemistry mapping. While limited genetic signals of parallel adaption to salinity changes between the two species are found from either genetic or gene expression variation perspective, a catalog of genes involved in ion transport, energy metabolism, structural reorganization, immune response, detoxification, and signal transduction is identified to be related to adaptation to salinity change in *Triplophysa* loaches. Together, our findings broaden our understanding of the population characteristics and adaptive mechanisms in sympatric Tibetan loach species and would contribute to biodiversity conservation and management of aquatic organisms on the QTP.

## Introduction

Aquatic organisms usually show population differentiation in accordance with the connectivity of water bodies. Therefore, for sympatric species, their genetic differentiation patterns and adaptive mechanisms to hydrological environments are expected to be similar unless affected by other factors (e.g., the degree of gene flow and effective population size difference). Freshwater organisms are vulnerable to drastic climate changes because they have limited abilities to disperse; and availability of water–their habitat–are highly climate-dependent (Sala et al., 2000; Dudgeon et al., 2006; Woodward et al., 2010; Poff et al., 2012; Tickner et al., 2020). The Qinghai–Tibet Plateau (QTP) is inevitably faced with the threat of climate change (Yao et al., 2012; Yao et al., 2019). For example, the mean air temperature has increased at a rate of 0.16–0.35 °C decade^−1^ from the early 1950s to 2014 on the QTP (Yao et al., 2012; Shen et al., 2015; Yao et al., 2019) in the context of global warming, which results in accelerated melting of glaciers and permafrost degeneration. To this end, the volume of many lakes on the QTP has increased rapidly in the past 40 years (Yang et al., 2017; Zhang et al., 2017; Zhang et al., 2020). Water storage of Lake Sêrling Co increased by 19–20 Gt from the 1970s to 2015 (Zhang et al., 2017). However, lakes in Southern Tibet (e.g., Lake Yumzhog Yumco) slightly decreased the water volume (Qiao et al., 2019). It suggests that water bodies on the QTP are susceptible to climate change. In addition, the salinity of lakes and rivers on the QTP varies widely, ranging from freshwater (e.g., Lake Mapam Yumco of 0.2 psu) to saltwater (e.g., Lake Lungmu Co of 138.6 psu) (Liu et al., 2021). The ongoing dramatic lake volume change may lead to rapid water salinity changes. For example, the salinity of Lake Sêrling Co has reduced from 18.5 g L^−1^ in 1979 to 12.4 g L^−1^ in 2017 (Zhu et al., 2019). Aquatic organisms on the QTP are thus facing challenges due to climate change.

*Triplophysa* loaches (Cypriniformes: Nemacheilidae), Schizothoracinae carps (Cypriniformes), and Sisoridae catfishes (Siluriformes) are the only native ichthyofauna on the QTP, with *Triplophysa* loaches being the largest component. *Triplophysa* loaches are mainly distributed on the QTP and its adjacent areas (Zhu, 1989). The genus *Triplophysa* originated ∼23.5 million years ago (Mya) (Wang et al., 2016) and rapidly diversified into ∼150 species accompanying the uplift of the QTP (Chen and Zhu, 1984; Chen et al., 1996; Fricke et al., 2019; GBIF, https://www.gbif.org/). *Triplophysa* loaches could be found in freshwater (e.g., River Lhasa He of 0.1 g L^−1^; Zhang, 2017) to saltwater (e.g., Lake Ngangla Ringco of 12.8 psu; Liu et al., 2021). For example, *T. stewarti* could be found in Lake Bam Co and Lake Sêrling Co with salinity exceeding the salinity tolerance reported in *Triplophysa* species (4.17 g L^−1^ in *T. yarkandensis* (Yao et al., 2018) and 3.74 g L^−1^ in *T. dalaica* (Wu et al., 2017). Earlier studies in *Triplophysa* loaches mainly focused on taxonomy (Zhu, 1989; Wu and Wu, 1992; Prokofiev, 2010; Kottelat, 2012), systematics (Wang et al., 2016; Chen et al., 2019; Wang T. et al., 2020), and biogeography (Chen et al., 1992; Wang et al., 2012; Feng et al., 2019; Hu et al., 2020). Next-generation sequencing (NGS) data are now largely adopted to identify molecular signatures of adaptation to high altitude (Wang et al., 2015a; Wang et al., 2015b), cave (Zhao et al., 2020), and salt/alkali (Chen et al., 2020) in *Triplophysa* loaches with comparative genomic approach, and several *Triplophysa* genomes have been determined with NGS data (Yang L. et al., 2019; Yang X. et al., 2019; Yuan et al., 2020; Zhou et al., 2021). However, the population genetics and adaptive mechanisms to salinity changes using NGS data have been rarely investigated in *Triplophysa* loaches (but see Chen et al., 2020; Hu et al., 2020; Yuan et al., 2020; Huo et al., 2022). How salinity changes caused by climate changes on the QTP may influence the biodiversity of *Triplophysa* loaches is still underexplored, especially at an intraspecific level.

Population genomics has been widely applied to wild populations. With either outlier analysis or environmental association analysis (EAA), it is possible to identify genetic variants underlying environmental adaptation (Guo et al., 2015; Guo et al., 2016; Ahrens et al., 2018; Liberles et al., 2020). However, these classical methods can only identify loci discretely. Linkage disequilibrium- (LD-) based analysis can screen for contiguous adaptive loci as a cluster and also possibly uncover interacting genetic elements. The LD-based analysis thus has facilitated our understanding of adaptive mechanisms in non-model organisms (Kemppainen et al., 2015; Li et al., 2018; Fang et al., 2020; McKinney et al., 2020). By conducting LD-based network analysis among loci, evolutionary phenomena, including chromosomal rearrangements, local adaptation, and geographic structure, which leave LD signals on genomes, can be identified (Kemppainen et al., 2015; McKinney et al., 2020). However, these earlier LD-based network methods can only handle tens of thousands of loci and thus are usually applicable to restriction-site-associated DNA (RAD) sequencing data or low-coverage Illumina sequencing data due to computational constraints (Kemppainen et al., 2015; McKinney et al., 2020). To deal with more loci, they have to incorporate stepwise dimension reduction (Li et al., 2018; Fang et al., 2020). Herein, we develop a novel sparse graph construction and learning based LD approach to identify the interconnection between multiple loci and cluster correlated loci with millions of loci. Our LD graph learning approach applied sparse high dimensional regression (Meinshausen and Bühlmann, 2006) to estimate the covariance structure among loci by estimating the skeleton of an undirected graph following the terminology of graph representation learning. Then, a graph community detection algorithm (Pons and Latapy, 2006) was used to cluster loci into LD blocks. These inferences were conducted first on loci within each chromosome and then on the genome-wide level. The advantages of our method include utilizing small-scale computational resources, the possibility of utilizing parallel computation, avoiding choosing arbitrary LD-related parameters (e.g., without splitting SNPs into non-overlapping windows, or specifying the LD thresholds to cluster loci (Fang et al., 2020)), and the capability of simultaneously inferring the LD structure among multiple loci without the need to calculate pairwise LD values.

In this study, we aim to investigate the population genetics and adaptive mechanisms to salinity changes in two *Triplophysa* loaches, *T. stewarti* and *T. stenura*, and are particularly interested in whether the two *Triplophysa* species show similar population differentiation patterns and common adaptive mechanisms to salinity changes. Therefore, we incorporated population genomic, transcriptomic, and electron probe microanalysis (EPMA) approaches. First, we characterized spatial-temporal habitat changes of the two *Triplophysa* species from sympatric populations using the otolith EPMA technique. Then, we generated single nucleotide polymorphism datasets including 70 individuals of seven populations from five localities of the two *Triplophysa* species to explore their population genetic features by utilizing whole-genome re-sequencing data and identified loci that bear similar signals of population genetic differentiation with a newly developed unsupervised LD graph learning method. Finally, we carried out RNA-seq in sympatric populations to investigate the transcriptomic responses to salinity changes in the two *Triplophysa* species.

## Materials and methods

### Sample collection

**FIGURE 1:**
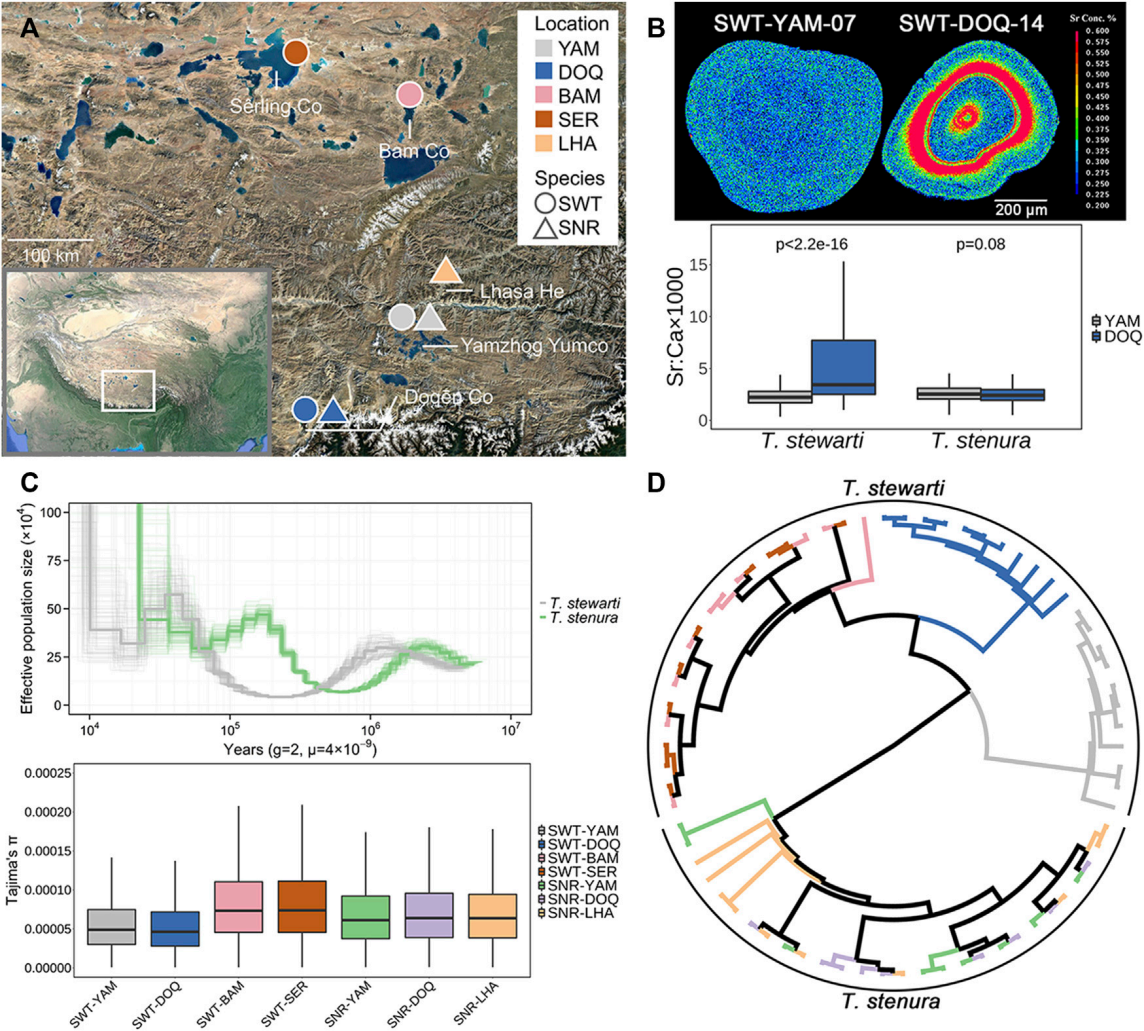
**(A)** Sampling sites in this study. SWT, *T. stewarti*; SNR, *T. stenura*; YAM, Lake Yamzhog Yumco; DOQ, Lake Doqēn Co; BAM, Lake Bam Co; SER, Lake Sêrling Co; LHA, River Lhasa He. **(B)** Top panel: two-dimensional imaging of Sr concentrations using otolith EPMA mapping analyses (SWT-YAM-07 and SWT-DOQ-14 taken as examples). Sr concentrations are represented by 16 colors, from red (highest) to green to blue (lowest). Bottom panel: comparisons of otolith Sr:Ca ratios along line transects from the core to the edge between Lake Yamzhog Yumco and Lake Doqēn Co populations of the two *Triplophysa* species. The significance test was complemented using the Wilcoxon Rank Sum Test with continuity correction. **(C)** Top panel: effective population size changes of *T. stewarti* and *T. stenura* estimated using PSMC. Thick lines represent population size inferences, and thin lines represent 100 bootstraps. Bottom panel: genetic diversity (Tajima’s π) of each population. **(D)** IQ-TREE reconstructed using the “common” SNP dataset. The color of each branch corresponds to the population in C (bottom panel).

The animal procedures were approved by the Animal Experiment Board of the Institute of Zoology, Chinese Academy of Sciences. Fish specimens were collected in June–July 2018 and May–June 2019 from five locations, including seven populations of the two *Triplophysa* species, *T. stewarti* and *T. stenura* (Figure 1A; Table 1). The base map in Figure 1A was generated using Google Earth Pro 7.3.3.7786 (2020 Google LLC). The fishes were captured with hand seines and/or minnow traps, temporally raised in drinking mineral water, and delivered to a laboratory in Beijing. Fishes for re-sequencing were kept in 95% ethanol, and fin clips were dissected for DNA extraction and sequencing. Fish gills for RNA sequencing were dissected and instantly frozen in liquid nitrogen.

**TABLE 1:** Sampling information of populations used in this study.

Species	Location	Population code	Coordinates	Number of individuals for DNA sequencing	Number of individuals for RNA sequencing	Number of individuals for otolith EPMA	Salinity (mg·L^−1^)
*T. stewarti*	Yamzhog Yumco	SWT-YAM	29.07°N, 90.39°E	11	7	8	1845.1^a^
*T. stewarti*	Doqēn Co	SWT-DOQ	28.22°N, 89.39°E	11	2	2	927^a^
*T. stewarti*	Sêrling Co	SWT-SER	31.67°N, 89.44°E	10	NA	NA	7662.84^a^
*T. stewarti*	Bam Co	SWT-BAM	31.35°N, 90.65°E	10	NA	NA	8233.89^a^
*T. stenura*	Yamzhog Yumco	SNR-YAM	29.07°N, 90.39°E	9	3	4	1845.1^a^
*T. stenura*	Doqēn Co	SNR-DOQ	28.22°N, 89.39°E	9	6	7	927^a^
*T. stenura*	Lhasa He	SNR-LHA	29.69°N, 90.89°E	10	NA	NA	100^b^

NA indicates that no individual is studied.

a Salinity of these locations was adopted from Yang (2018).

b Salinity of River Lhasa He was adopted from Zhang (2017).

### Electron probe microanalysis

The asteriscus otoliths were extracted from 21 individuals, including 18 individuals used in RNA-seq (Table 1). The experimental procedures followed the study of Liu et al. (2020). The extracted otoliths were embedded in epoxy resin (Epofix, Struers, Copenhagen, Denmark), mounted on a glass slide, and ground to expose the core using a grinding machine (70 μm/35 μm, Discoplan-TS, Struers, Copenhagen, Denmark). The otoliths were further polished on an automated polishing wheel (LaboPol-35, Struers, Copenhagen, Denmark). After polishing, the otoliths were cleaned in an ultrasonic bath and rinsed with Milli-Q water. Finally, the otoliths were dried and carbon-coated using a high vacuum evaporator (JEE-420, JEOL Ltd., Tokyo, Japan) before the EPMA examination. The line transect analyses, which measured Sr and Ca concentrations along the longest axis from the core to the edge of the otoliths, as well as the mapping analyses, which measured Sr concentrations evenly on the whole cross section, were complemented using a wavelength dispersive X-ray electron microprobe (JXA-8100, JEOL Ltd., Tokyo, Japan). Strontium titanate (SrTiO_3_) and calcium carbonate (CaCO_3_) were used as standards. For the line transect analyses, the accelerating voltage, beam current, and counting time were 15 kV, 2 × 10^−8^ A, and 15 s, respectively. The electron beam was 2 μm in diameter, spaced at 4 μm intervals. For the mapping analyses, the accelerating voltage, beam current, and counting time were 15 kV, 5 × 10^−7^ A, and 30 ms, with the electron beam of 2 μm in diameter. The pixel size for the mapping analyses was 3 × 3 μm.

### DNA and RNA sequencing

DNA and RNA extraction, library construction, and sequencing were done by Novogene Technology Co., Ltd. Briefly, DNA was extracted from the fin clips and then sheared into short fragments by ultrasonication. Short DNA fragments were end-repaired, phosphorylated, A-tailed, and ligated to index adapters. After purification and PCR amplification, the constructed libraries were sequenced on the Illumina HiSeq platform with a 150 bp paired-end strategy. In total, 408.84 G of raw data of 70 individuals from the seven populations were produced. Low-quality reads were removed according to the following criteria: 1) paired reads were removed when containing adapters; 2) paired reads were removed if the N content of either single-end read exceeds 10% of the total length; and 3) paired reads were removed if the number of low-quality bases (Q ≤ 5) of either single-end read exceeds 50% of the total length. Read quality control was double-checked using FastQC v0.11.9. Finally, 408.01 G of clean genomic data were obtained (Supplementary Table S1).

Total RNA was first extracted, and magnetic beads with oligo (dT) were used for enriching mRNA. Then, the mRNA was fragmented into short fragments. cDNA was synthesized using these mRNA fragments as templates and random hexamers as primers. The short cDNA fragments were purified, end-repaired, A-tailed, and then ligated to index adaptors. Fragments of 250–300 bp were selected for PCR amplification, purification, and cDNA library construction. These libraries were sequenced on Illumina NovaSeq 6000 platform with a 150 bp paired-end strategy. In total, 299.6 million raw reads of 18 transcriptomes from four populations were obtained (Supplementary Table S2). The number of clean reads for each transcriptome ranged from 11.1 and 19.8 million (Supplementary Table S2), and 286.0 million clean reads were obtained after quality filtering following the criteria of DNA read filtering.

### SNP calling

The *T. tibetana* genome was retrieved from GenBank (accession number SOYY00000000; Yang X. et al., 2019) and used as the reference. The reference genome was first indexed using the “bwa index” in BWA Version 0.7.17-r1188 (Li, 2013), and then re-sequencing clean reads were aligned to the reference using “bwa mem.” SAMtools (Version 1.8) (Li et al., 2009; Danecek et al., 2021) were used to convert SAM to BAM format, remove PCR duplicate reads, and calculate mapping rate and depth of coverage. The percentage of reads aligned to the reference genome ranged from 74.80% to 87.74%, and the depth of coverage was 4.53 × to 7.08 × (Supplementary Table S1).

Raw SNPs were obtained using BCFtools Version 1.8 (Li, 2011; Danecek et al., 2014; Danecek et al., 2021) commands “bcftools mpileup -C 50 -d 10,000 -q 20 -Q 20” and “bcftools call -m.” SNPs were further filtered using “bcftools filter” in BCFtools and VCFtools Version 0.1.13 (Danecek et al., 2011), using the following criteria: 1) SNPs within 10 bp of an indel were removed; 2) only bialleles were retained; 3) only genotypes with a minimum depth of 2 and a maximum depth of 20 were retained; 4) SNPs with a proportion of missing data >50% across all populations and within each population were removed; 5) only SNPs with quality value ≥25 were retained; and 6) only SNPs with a minor allele frequency ≥0.05 were retained.

With the abovementioned criteria, an SNP dataset, hereinafter referred to as the “common” SNPs, was generated by including all 70 individuals from the two *Triplophysa* species, as well as “individual” SNP datasets for each of the two *Triplophysa* species. The “common” SNP dataset consists of 590,335 SNPs with 589,179 SNPs on 25 chromosomes and 1,156 SNPs on 267 scaffolds. The “individual” SNP dataset for *T. stewarti* contained 351,745 SNPs with 350,930 SNPs on 25 chromosomes and 815 SNPs on 267 scaffolds, and the SNP dataset for *T. stenura* contained 235,689 SNPs with 234,682 SNPs on 25 chromosomes and 1,007 SNPs on 267 scaffolds, respectively. The number of shared SNPs between the two “individual” SNP datasets was 18,428.

### Effective population size, genetic diversity, and population structure analysis

In order to estimate effective population size changes in the two species, two individuals of SWT-YAM-10 and SNR-YAM-03 were further DNA-sequenced to ∼100 × depth of coverage. Read mapping, sorting, and PCR duplicates removing were complemented, as shown in the above section. Effective population size changes were estimated using Pairwise Sequentially Markovian Coalescent (PSMC) Version 0.6.5-r67 (Li and Durbin, 2011). Consensus calling was done using “samtools mpileup -C50 -Q20 -q20 -d10000,” “bcftools view -c,” and “vcfutils.pl vcf2fq -d20 -D120 -Q20.” Fastq format was converted to a psmc input file format using fq2psmcfa. PSMC was run with options of -N25 -t15 -r5 -p “4+25*2+4+6”. Bootstrap was conducted by randomly sampling with replacement from 500 kb sequence segments generated by the splitfa tool, and 100 rounds were performed. The effective population size changes were calculated with the generation time of 2 years and 4 × 10^−9^ substitutions per synonymous site per year following the study on *T. bleekeri* (Yuan et al., 2020) and visualized using psmc_plot.pl and R package “ggplot2” v3.3.2 (Wickham, 2016).

Non-overlapping 100 kb sliding window Tajima’s π was calculated based on “individual” SNP datasets using VCFtools to estimate genetic diversity in each population. To investigate genome-wide differentiation in each species, Weir and Cockerham’s *F*_ST_ in non-overlapping 100 kb sliding windows between populations was calculated by VCFtools using “individual” SNP datasets. Windows with 15–205 SNPs, comprising ∼90% of genome-wide windows, were included in genetic diversity and *F*_ST_ calculations. Principal component analysis (PCA) was performed using PLINK Version 1.90b6.18 (Chang et al., 2015; Purcell and Chang, 2020) with the “common” dataset and two “individual” datasets, respectively. Population stratification and genetic admixture were inferred based on “individual” SNP datasets of the two species using ADMIXTURE v1.3.0 (Alexander et al., 2009). Genetic clusters (K) ranging from 1 to *n* + 1 (*n* is the number of populations in the dataset) were applied. The optimal K was determined through a cross-validation procedure (Alexander and Lange, 2011). Maximum-likelihood trees were reconstructed using IQ-TREE multicore Version 2.0.6 (Nguyen et al., 2014; Minh et al., 2020), with 1,000 ultrafast bootstrap replicates to determine branch confidence values (Hoang et al., 2017). The best-fitting model was estimated using ModelFinder and selected based on the corrected Akaike information criterion (cAIC) (Kalyaanamoorthy et al., 2017).

### Identifying genetic differentiation with LD-based analyses

An unsupervised linkage disequilibrium graph learning method (hereinafter referred to as LD graph learning) was developed to identify loci showing similar genetic differentiation signals. The object of LD graph learning is first to build an undirected correlation network among SNPs on the basis of their pairwise LDs and then use a graph-based clustering approach to classify SNPs into LD blocks. The proposed LD graph learning comprises two steps: chromosome-wise LD network construction and genome-wide LD network construction.

First, the local LD network was constructed on the basis of SNPs separately in each chromosome using an adapted version of the sparse high dimensional graph estimation approach introduced in Meinshausen and Bühlmann (2006). In turn, each SNP *j* (*j* = 1, .., *p*_*c*_; *p*_*c*_ is the total number of the SNPs in the chromosome *c*) is considered a response variable, and all the SNPs other than SNP *j* are considered explanatory variables in a multinomial LASSO regression model (Friedman et al., 2010), defined as

$$\underset{\beta_{k}}{\max}\left[\frac{1}{n}\sum_{i=1}^{n}y_{ik}\log p\left(x_{ij}=k|x_{il}\right)-\lambda \sum_{l\neq j}\left|\beta_{kl}\right|\right]$$ (1)

with $p\left(x_{ij}=k|x_{il}\right)=\frac{\exp\left(\sum_{l\neq j}x_{il}\beta_{kl}\right)}{\sum_{s\in \left\{0,1,2\right\}}\exp\left(\sum_{l\neq j}x_{il}\beta_{sl}\right)}$ ,where *x*_*ij*_ is the genotype value of individual *i* and SNP *j*, coded as *x*_*ij*_ = 0, 1, 2 for genotypes AA, AB, and BB, respectively. *y*_*ik*_ is an indicator variable: if *x*_*ij*_ = *k* (*k* = 0, 1, or 2), *y*_*ik*_ = 1; otherwise, *y*_*ik*_ = 0. $\beta_{kl}$ is the regression parameter of SNP *x*_*il*_, $\lambda \sum_{l\neq j}\left|\beta_{kl}\right|$ is a penalty term that can shrink many of the regression parameters to zero, when there is no correlation between these SNPs and the SNP *j*, and *λ* is a shrinkage factor that decides the number of SNPs to have zero regression coefficients. In practice, Equation 1 was solved by the coordinate descent algorithm, implemented in the R package “glmnet” (Friedman et al., 2010). The optimal value of the shrinkage factor *λ* was determined by maximizing the Akaike information criterion (AIC). From Equation 1, the non-zero regression coefficients $\sqrt{\sum_{k\in \left(0,1,2\right)}\beta_{kl}^{2}}>0$ indicate that the SNP *j* and *l* are in LD with each other. If SNP *j* and *l* are presented as two nodes in an undirected graph, there is an edge between *j* and *l*. Consequently, on the basis of the output of Equation 1, we can generate an undirected and unweighted graph, which can be represented by a *p*_*c*_ × *p*_*c*_ adjacency matrix *A*, with element *A*_*lj*_ = 1 indicating SNP *l* and *j* are in LD and *A*_*lj*_ = 0 indicating *l* and *j* are not in LD. The graph is expected to be sparse, meaning that most of the elements of *A* equal zero. We then applied a random-walk-based clustering approach (Pons and Latapy, 2006) to define LD blocks, using the function “cluster_walktrap” in the R package “igraph” (Csardi and Nepusz, 2006).

Next was to construct the genome-wide network based on the chromosome-wise clusters. To reduce the dimension of the data, in each chromosome-wise cluster, we calculated the principal components of the SNPs within that cluster and then took the first PCs that accumulatively explained 80% of the genetic variance to be used as covariates in the genome-wide LD network analysis. Considering each chromosome-wise cluster *C*_*j*_ (*j* = 1, … , *M*) as the basic unit (like “SNP”), a group LASSO regression (Yuan and Lin, 2006) was then applied to detect the correlation between each cluster *j* and all other clusters as

$$\underset{\alpha_{j}}{\min}\frac{1}{2n}{\left(z_{ij1}-\sum_{l\neq j}\sum_{k=1}^{m_{j}}z_{ilk}\alpha_{lk}\right)}^{2}+\lambda \sum_{l\neq j}{\left\|\sum_{k=1}^{m_{j}}\alpha_{lk}^{2}\right\|}_{2}$$ (2)

where *z*_*ij*1_ (*j* = 1, … , *M*; *M* is the total number of chromosome-wise clusters) represents the first PC within the cluster *j*, *z*_*ilk*_ (*l* = 1, … , *m*_*l*_; *m*_*l*_ is the total number of PCs in cluster *l*) are the PCs defined in clusters other than *j*, *α*_*lk*_ are the regression coefficients that define the level of correlation between the first PC in cluster *j* and all the PCs in cluster *l*, and $\lambda \sum_{l\neq j}{\left\|\sum_{k=1}^{m_{j}}\alpha_{lk}^{2}\right\|}_{2}=\lambda \sum_{l\neq j}\sqrt{\sum_{k=1}^{m_{j}}\alpha_{lk}^{2}}$ is the group LASSO penalty, and it can shrink a group of regression parameters defined within a cluster to zero if that cluster was not correlated with the cluster *j*. The optimal shrinkage was again decided by AIC. Here, algorithm (2) generates a sparse undirected and unweighted graph of *M* nodes representing *M* chromosome-wise clusters, and the edge between a pair of clusters tells whether the two are correlated with each other. The genome-wide SNP clusters were then obtained using the same random-walk-based clustering approach as in chromosome-wise LD network construction.

To further validate the robustness of our LD graph learning approach in detecting clusters of highly differentiated SNPs, an earlier developed LD-based network analysis, three-step LDna (Kemppainen et al., 2015; Fang et al., 2020), was used. Three-step LDna was performed following the procedures described in Fang et al. (2020). In brief, pairwise LD (measured by *r* ^2^ statistic) between SNPs was first calculated using ngsLD v1.1.1 (Fox et al., 2019) within the maximum distance of 100 SNPs between any two SNPs. SNPs were then divided into non-overlapping windows within each chromosome using the “LDnClusteringEL” function in LDna package v0.65 (Kemppainen et al., 2015; Li et al., 2018). The desired number of SNPs per window (“nSNPs” parameter) was set as 800 for *T. stewarti* populations and 1,000 for *T. stenura* populations, with other parameters of w1 = 10, LD_threshold1 = 0.5, and LD_threshold2 = 0.8. Maximally connected loci (MCL) that exhibited the highest median LD with all other SNPs in the same window were chosen. Next, MCL were used to perform LDna within each chromosome. The parameters were set to *λ*_*lim*_ = 8 and |*E*|_*min*_ = 50 to extract single outlier clusters (SOCs). Finally, only MCL in each SOC was selected to perform genome-wide LDna. SOCs were grouped into genome-wide LD clusters, and original SNPs were extracted. In *T. stenura*, only 4.513 windows were obtained in LDnClusteringEL, so genome-wide LDna was directly performed using the MCL of these 4,513 windows. Pairwise *F*_ST_ per site of these LD clusters was calculated using VCFtools. Absolute differentiation *D*_xy_ was calculated using the script popgenWindows.py (https://github.com/simonhmartin/genomics_general/blob/master/popgenWindows.py).

In order to identify genetic variation associated with salinity variation in each of the two *Triplophysa* species, both the LD graph learning and three-step LDna were performed with “individual” SNP datasets by considering different scenarios of parallel adaptation according to salinity in population locations (Figure 1A; Table 1). Seven populations from the five water bodies of the two species were thus classified as a low-salinity group of River Lhasa He population, mid-salinity group of Lake Yamzhog Yumco and Lake Doqēn Co populations, and high-salinity group of Lake Bam Co and Lake Sêrling Co populations, which was used in the LD graph learning and three-step LDna for adaptation signal detection.

### Environmental association analysis

BAYENV2 (Coop et al., 2010; Günther and Coop, 2013) was used to analyze the association between genetic variation and salinity variation with “individual” SNP datasets. The two “individual” datasets were first filtered to estimate the null model, allowing no missing data and limited LD (*r* ^2^ < 0.2). SNPs showing the maximum/minimum 5% of average *F*_ST_, which might indicate a directional or balancing selection, were also removed. The remaining neutral SNPs (9,448 SNPs in *T. stewarti* and 38,047 SNPs in *T. stenura*) were used to estimate covariance matrices from 100,000 iterations. Covariance matrix estimation was independently run three times. By using the mean matrix as the null model and original “individual” SNP datasets, correlations between genetic variation and salinity variation were detected. BAYENV2 was independently run three times using different random seeds for 50,000 iterations. Bayes factors were averaged across the three independent runs. SNPs associated with salinity adaptation using the above unsupervised and supervised methods were annotated using ANNOVAR (version date 7/6/2020; Yang and Wang, 2015).

### Differential gene expression analysis

RNA-seq reads were aligned to the reference genome using HISAT2 version 2.2.1 (Kim et al., 2019). 31.53%–67.76% of reads were aligned to the reference genome (Supplementary Table S2). Next, transcripts were assembled and quantified for each transcriptome using StringTie v2.1.4 (Pertea et al., 2015; Pertea et al., 2016; Kovaka et al., 2019) with default parameters and with the reference annotation guiding the assembly processes. The assembled transcripts for each sample were then merged, and the merged transcripts were used to estimate transcript abundances for each sample. The “prepDE.py” script in the StringTie package was used to create a gene count matrix as input for differential expression analysis.

PCR duplicate reads in BAM files were removed using SAMtools. SNP calling and filtering were complemented using BCFtools and VCFtools with parameters the same as in re-sequencing data analysis. The filtering criteria were the same as described in the “SNP filtering” section, except that genotypes with depth >20 were also retained and SNPs with a proportion of missing data >20% across all populations were removed. PCA was performed using PLINK.

Differential expression analysis based on the negative binomial distribution was done by R package “DESeq2” v1.26.0 (Love et al., 2014). The DESeq2 model was corrected internally for library size differences. In order to avoid the distance measure being dominated by a few highly variable genes, rlog-transformed data were used to calculate Euclidean distance between samples. The significance level was adjusted with the Benjamini and Hochberg correction (Benjamini and Hochberg, 1995) to control for the false discovery rate in multiple pairwise comparisons. Only genes with raw count >0 for each sample of each species were kept for extracting differential expressed genes to avoid the impacts of a relatively low depth of coverage. Expression was deemed significantly different for genes with a corrected *p*-value below 0.05.

## Results

### Ecological and population genetic characteristics

Microchemical mapping with EPMA showed that *T. stewarti* from Lake Doqēn Co has a significantly higher Sr:Ca ratio in otolith than *T. stewarti* from Lake Yamzhog Yumco (the Wilcoxon Rank Sum Test, *p* < 2.2e-16), whereas the Sr:Ca ratio is not significantly different between populations of *T. stenura* (the Wilcoxon Rank Sum Test, *p* = 0.08). The Sr:Ca ratio in otolith is significantly different between *T. stewarti* and *T. stenura* populations either from different lakes or from the same lakes (the Wilcoxon Rank Sum Tests, *p* < 0.001) (Figure 1B). PSMC showed that both species in Lake Yamzhog Yumco experienced population contraction from about 1–2 Mya, though *T. stenura* population expanded to 5×10^5^ at 200 Kya, whereas *T. stewarti* maintained a relatively low effective population size at the same period (Figure 1C). Population genetic diversity was lower in populations SWT-DOQ and SWT-YAM compared to SNR-DOQ and SNR-YAM, respectively (paired *t*-tests, *p* < 2.2e-16; Figure 1C). Population genetic relationships were inferred with the “common” and “individual” SNP datasets obtained. PCA and phylogenetic inference with the “common” dataset showed that the two species were explicitly separated into two clusters (Supplementary Figure S1 and Figure 1D). The four *T. stewarti* populations were grouped into three clusters, with two populations (SWT-BAM and SWT-SER) in one cluster. The three populations of *T. stenura* were mixed together (Figure 1D and Supplementary Figure S1). Bayesian clustering inference in ADMIXTURE and pairwise *F*_ST_ of “individual” SNP datasets also supported the above pattern of population differentiation, with *T. stewarti* populations deeply diverged but *T. stenura* populations unseparated (Supplementary Figure S1, Supplementary Figures S2, S3).

### Genetic differentiation associated with salinity variation

**FIGURE 2:**
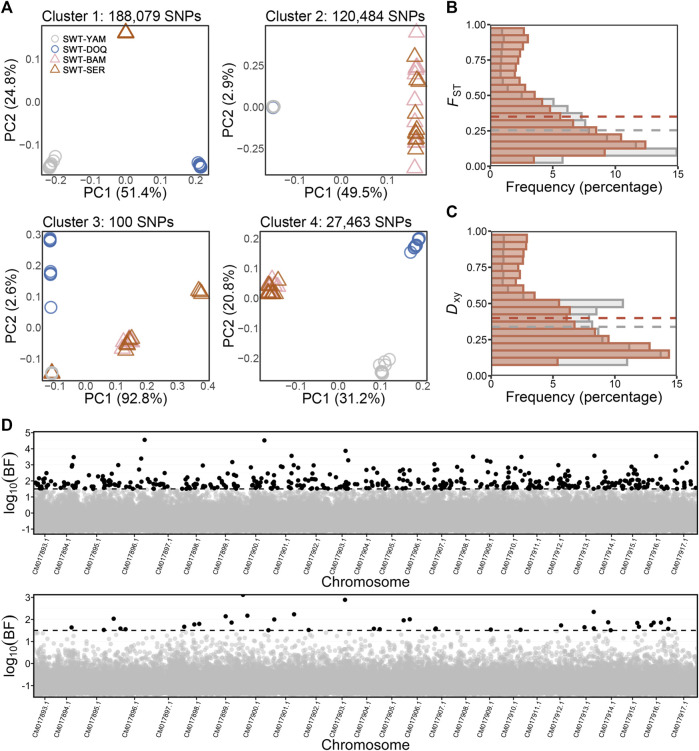
**(A)** PCA of genome-wide SNP clusters identified using the LD graph learning method in all four populations of *T. stewarti*, with cluster 2 showing the pattern of parallel adaptation to salinity between high- (triangles) and mid-salinity (circles) groups. **(B,C)** Distributions of fixation index *F*_ST_ **(B)** and absolute population differentiation *D*_xy_ **(C)** of SNPs in genome-wide SNP cluster 2 (red bars) compared with all SNPs (gray bars) between high- and mid-salinity groups. Red and gray dash lines represent the average *F*_ST_/*D*_xy_ of SNPs in genome-wide SNP cluster 2 and *F*_ST_/*D*_xy_ of all SNPs, respectively. **(D)** Environmental association analysis of *T. stewarti* (upper panel) and *T. stenura* (lower panel). SNPs with a Bayesian factor above the threshold (log_10_(BF) = 1.5, dash line) are colored in black.

Using the newly developed unsupervised LD graph learning approach, four genome-wide SNP clusters were identified with an “individual” SNP dataset of the 350,930 SNPs for the four *T. stewarti* populations, of which the genome-wide SNP cluster 2 containing 120,484 SNPs showed clear genetic differentiation between high- and mid-salinity groups (Figure 2A). SNPs in cluster 2 showed significantly higher differentiation than all SNPs between the two salinity groups according to *F*_ST_ and *D*_xy_ values (Figures 2B, C). A total of 45 out of 71 chromosome-wide SNP clusters within this genome-wide SNP cluster 2, composed of 63,344 SNPs, showed higher weighted *F*_ST_ than all SNPs between high- and mid-salinity groups. The newly developed unsupervised LD graph learning approach was also performed in pairwise high- and mid-salinity *T. stewarti* populations. A total of 350 genome-wide SNP clusters were identified in comparison between SWT-SER and SWT-YAM, 311 in comparison between SWT-SER and SWT-DOQ, 275 in SWT-BAM and SWT-YAM, and 258 in SWT-BAM and SWT-DOQ, of which 15, 10, 14, and 11 genome-wide SNP clusters showed higher *F*_ST_ than weighted *F*_ST_ calculated using all SNPs between the two populations, respectively. 1,640 SNPs were found in these highly differentiated genome-wide SNP clusters in all the four *T. stewarti* population pairs (Supplementary Figure S3). Of these 1,640 SNPs, 1,615 SNPs (98.5%) were included in genome-wide SNP cluster 2 when LD graph learning was done with all of the four *T. stewarti* populations, and 1,583 SNPs (96.5%) were in 45 highly differentiated chromosome-wide clusters of genome-wide cluster 2. The unsupervised three-step LDna with the “individual” SNP dataset of the 350,930 SNPs in the four *T. stewarti* populations identified 11 LD clusters, and LD cluster 2, containing 20,311 SNPs, showed similar genetic signatures between high- and mid-salinity populations as the genome-wide SNP cluster 2 identified by LD graph learning (Supplementary Figures S3, S4). 18,545 out of 20,311 SNPs (91.3%) in this LD cluster 2 identified by three-step LDna were also included in the genome-wide SNP cluster 2 identified by LD graph learning. Using these abovementioned unsupervised methods, we identified 1,443 common SNPs showing high genetic differentiation between salinity groups. Furthermore, the supervised EAA method with BAYENV2 identified 410 SNPs, which were strongly associated with salinity variation, and 20 of the 410 SNPs were highly differentiated loci and associated with salinity variation with the abovementioned unsupervised methods (Figure 2D and Supplementary Figure S3). These 20 SNPs were annotated within or close to genes that function in response to osmotic stress or salinity acclimation, for example, hepatocyte nuclear factor 4-alpha (*HNF4A*), facilitated glucose transporter member 1 (*GLUT1*), gamma-glutamyl hydrolase (*GGH*), and dipeptidyl aminopeptidase-like protein 6 (*DPP6*) (Supplementary Table S4).

The newly developed unsupervised LD graph learning, three-step LDna, and supervised EAA with BAYENV2 were also carried out to identify genetic differentiation associated with salinity variation in *T. stenura*. Because PCA of genome-wide SNP clusters did not separate the low-salinity group (SNR-LHA) from the mid-salinity group (SNR-YAM and SNR-DOQ), *F*_ST_ instead of PCA was used as a benchmark in LD graph learning. 142 out of 368 genome-wide SNP clusters containing 77,730 SNPs showed higher weighted *F*_ST_ than those of all SNPs. Totally, 61,858 SNPs were commonly found to be in highly differentiated genome-wide clusters in LD graph learning in the two pairwise *T. stenura* populations, SNR-YAM and SNR-LHA, as well as SNR-DOQ and SNR-LHA. Moreover, 22,213 SNPs were commonly found to be in highly differentiated genome-wide SNP clusters when LD graph learning was performed with all populations and between population pairs in *T. stenura*. Using three-step LDna, 16 SOCs containing 2,265 SNPs were identified (Supplementary Figure S5). A total of 1,122 SNPs in six SOCs out of these 2,265 SNPs showed higher genetic differentiation, of which 433 SNPs overlapped with the 22,213 SNPs identified using LD graph learning. BAYENV2 detected 38 SNPs strongly associated with salinity in *T. stenura*, with only one SNP overlapping with SNPs identified using the above-unsupervised methods of LD graph learning and three-step LDna (Figure 2D). No common SNP showing genetic differentiation associated with salinity variation was identified between the two species *T. stewarti* and *T. stenura*.

### Transcriptome responses to salinity change

**FIGURE 3:**
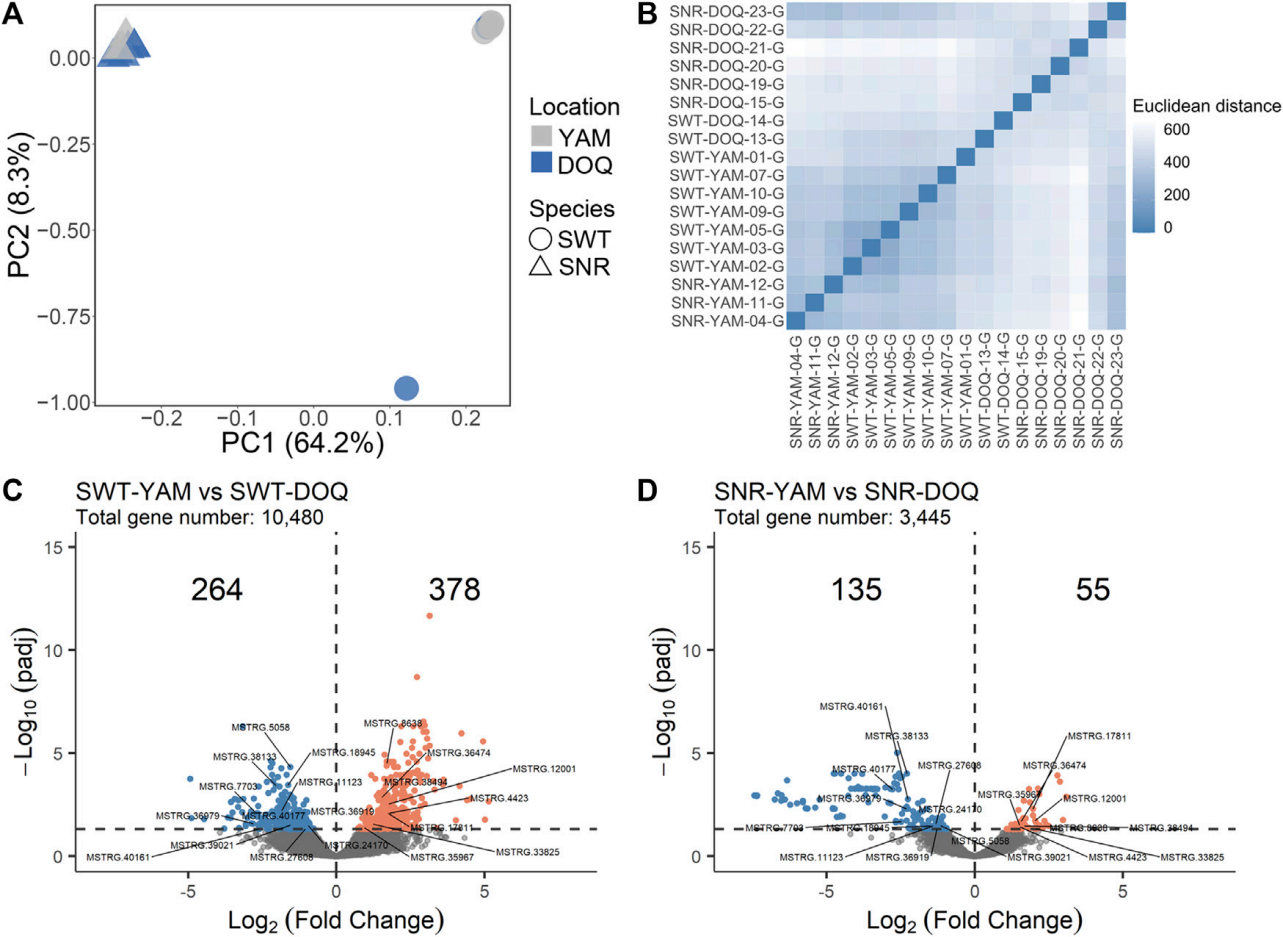
**(A)** PCA of four populations using SNPs called from RNA-seq data. **(B)** Euclidean distances among RNA-seq samples calculated using the gene count matrix after DESeq2 correction for library differences and rlog-transformation. **(C,D)** Volcano plots showing DEGs between Lake Yamzhog Yumco and Lake Doqēn Co populations of *T. stewarti* **(C)** and *T. stenura* **(D)**. The number of genes upregulated (orange dots) and downregulated (blue dots) in Lake Yamzhog Yumco populations are marked. The 20 gene IDs labeled are common DEGs between the two species.

The two species of *T. stewarti* and *T. stenura* were separated by the first PC based on 8,025 SNPs from RNA-seq data (Figure 3A). The expression profiles of samples from the same location showed higher similarity (Figure 3B); that is, Euclidean distances among Lake Yamzhog Yumco samples were closer compared to those between samples from Lake Yumzhog Yumco and Lake Doqēn Co., and the same for Lake Doqēn Co samples. In *T. stewarti*, 642 out of 10,480 genes were identified as differentially expressed genes (DEGs) between population SWT-YAM and SWT-DOQ, with 264 downregulated and 378 upregulated in the Lake Yamzhog Yumco population (Figure 3C). In *T. stenura*, 3,445 genes remained after filtering, and 190 genes were identified as DEGs between population SNR-YAM and SNR-DOQ, with 135 downregulated and 55 upregulated genes in the Lake Yamzhog Yumco population (Figure 3D). Twenty genes were identified as common DEGs in both species (Figures 3C,D). Of these 20 genes, eight were novel genes assembled by StringTie and 12 genes were genes with annotations, including peroxiredoxin-1 (*PRDX1*), C member 5 multi-specific organic anion transporter (*ABCC5*), and adenomatous polyposis coli protein (*APC*).

## Discussion

The most salient finding in this study is that the two sympatric and phylogenetically closely related Tibetan loach species show contrasting population differentiation patterns, which broaden our understanding of the Tibetan loach diversification on the QTP. In addition, a catalog of genes involved in ion transport, energy metabolism, structural reorganization, immune response, detoxification, and signal transduction is found to be related to adaptation to salinity change in *Triplophysa* loaches, whereas the two phylogenetically closely related Tibetan loach species show limited genetic signals of parallel adaption to salinity changes from either genetic or gene expression variation perspective. We will discuss these findings in detail below. Finally, we highlight the utilization of our newly developed unsupervised LD graph learning approach with large SNP datasets in the molecular ecological study of non-model organisms.

### Causes of contrasting population differentiation

Phylogeography provides the key to understanding the evolutionary history of organisms and responses of populations and species to geological events (Schluter, 2000). The phylogeography of *Triplophysa* loaches on the QTP is thought to result from the occurrence of geological events associated with the QTP uplift (Wang et al., 2016). Based on millions of genome-wide SNPs, *T. stewarti* populations are deeply diverged into three clusters of the BAM/SER populations, the YAM population, and the DOQ population (Figure 1D), which may be attributed to geographical barriers. The Gandisê Mountains and the Nyainqêntanglha Mountains divided these lakes and rivers into the northern part (including Lake Sêrling Co and Lake Bam Co) and the southern part (including Lake Yamzhog Yumco, Lake Doqēn Co, and River Lhasa He). These two parts were once connected when the Qiangtang Palaeolake, merging Sêrling Co and Bam Co together, outflew into the external drainage system of southern Tibet during 40–35 ka BP, but then disconnected due to lake level decrease (Zhao et al., 2002a; Zhao et al., 2002b). As a result, the geographical barriers facilitate the genetic divergence between the northern (BAM/SER) and southern (YAM and DOQ) populations in *T. stewarti*. As lake volume in the northern part is rapidly increasing in the past 40 years, the genetic mixture between BAM and SER populations in *T. stewarti* suggests that the drainage is still connected between Sêrling Co and Bam Co nowadays. Interestingly, the three *T. stenura* populations are genetically mixed, which is different from sympatrically distributed *T. stewarti* populations in Yamzhog Yumco and Doqēn Co (Figure 1D). The difference between sympatric populations in the two species of Tibetan loaches is also seen in trajectories of changes in effective population size. It showed that *T. stewarti* had experienced a recent population contraction; meanwhile, *T. stenura* experienced population expansion in Yamzhog Yumco, and as such, a lower genetic diversity is found in *T. stewarti* than that in *T. stenura* in Yamzhog Yumco (Figure 1C). In addition, otolith Sr:Ca ratio EPMA showed that sympatric populations in the two species possess different ecological characteristics. Otolith Sr:Ca ratio EPMA reflects the spatial-temporal habitat changes in fish (Campana, 1999; Secor and Rooker, 2000; Yang et al., 2006). Thus, genetic divergence patterns in sympatric populations in the two species are in accordance with their ecological divergence patterns. Furthermore, according to Wu and Wu (1992), *T. stewarti* and *T. stenura* possess different morphs of the posterior chamber of the swim bladder. The posterior chamber of the swim bladder in *T. stewarti* is well developed but degenerated in *T. stenura*. With a well-developed swim bladder, *T. stewarti* might prefer still water or argodromile, whereas *T. stenura* prefers flowing water or riffle streams. *T. stewarti* thus may possess restricted dispersal capability compared with *T. stenura*. Therefore, the morphological difference in the posterior chamber of the swim bladder between the two *Triplophysa* loaches might explain their explicit ecological and genetic differentiation between sympatric populations. Herein, we further confirmed that the deeply diverged *T. stewarti* populations belong to one taxonomic species rather than different ones. According to Wu and Wu (1992) and Zhu (1989), *Triplophysa* species (*T. brevicauda*, *T. leptosoma*, *T. microps*, *T. stoliczkai*, *T. stenura*, and *T. orientalis*), showing sympatric distributions with our sampling sites, are morphologically distinct to *T. stewarti*, with caudal peduncle laterally compressed and/or swim bladder reduced, whereas *T. stewarti* showed more slender caudal peduncle with roughly round-shaped cross-section and developed posterior chamber of the swim bladder. Moreover, the genetic distance among *T. stewarti* populations measured by Kimura 2-parameter (K2P) distance of mitochondrial *COI* gene sequences ranged from 0.0013 to 0.0170, within the range of intraspecific genetic distance in *Triplophysa* (Wang T. et al., 2020). In fact, many species in *Triplophysa* loaches are distributed in sympatry as the two species *T. stewarti* and *T. stenura* in our study, with the posterior chamber of the swim bladder well developed in one species but reduced in another (Zhu, 1989; Wu and Wu, 1992). It seems that divergence in functional traits, such as swim bladder, might have played an important role in *Triplophysa* diversification along with geological events.

### Genetic mechanisms underlying adaptation to salinity change

Lakes and rivers on the QTP exhibit large salinity heterogeneity (Liu et al., 2021). Worldwide climate change has recently led to drastic lake volume changes on the QTP (Yang et al., 2017; Zhang et al., 2017; Zhang et al., 2020), which may result in continuous salinity changes in these lakes. Thus, adaptation to salinity changes is key to the biodiversity evolving in aquatic organisms on the QTP. In this study, we investigated the genetic mechanisms of salinity adaptation in the two *Triplophysa* species: *T. stewarti* and *T. stenura*. First, the two *Triplophysa* species showed limited genetic signals of parallel adaption to salinity changes from either genetic or gene expression variation. A total of 39 genes were identified to harbor highly differentiated loci associated with adaptation to salinity changes in both *T. stewarti* and *T. stenura* with millions of genome-wide SNPs using unsupervised genetic differentiation methods. With RNA-seq data, 20 common DEGs in gill were identified between *T. stewarti* and *T. stenura*, of which few seem to be associated with salinity adaptation (Figures 3C,D). In fact, non-parallel genetic changes more frequently occur at the interspecific level, especially with adaptation to given selection pressure involving polygenes, such as adaptation to salinity change in fishes (Wang and Guo, 2019), although parallelism often underlies adaptation to a similar environment (Stern, 2013). For example, genetic changes associated with repeated marine-freshwater divergence in two geographically coexisting and ecologically similar stickleback species, nine- and three-spined sticklebacks, are largely non-parallel (Wang Y. et al., 2020; Kemppainen et al., 2021). Second, we noticed that prevalent genetic changes associated with salinity change were identified in *T. stewarti* but few in *T. stenura* (Figure 2D), which might be due to *T. stewarti* having populations adapted to Lake Bam Co and Lake Sêrling Co with ∼8 mg L^−1^ of salinity and high genetic connectivity among *T. stenura* populations (Figure 1D). It suggests that *T. stewarti* is a better model for understanding genetic mechanisms of adaptation to salinity change on the QTP.

**FIGURE 4:**
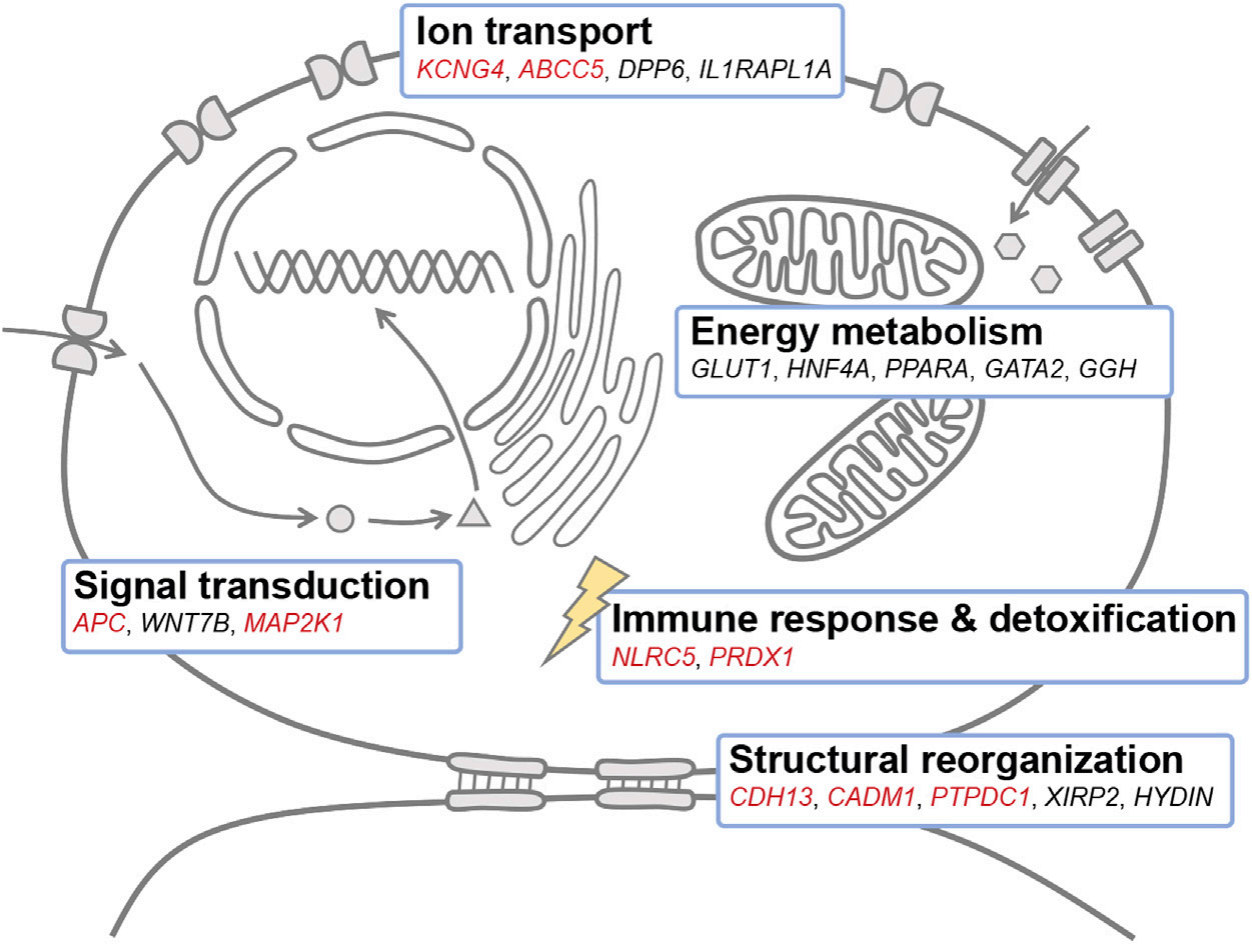
Candidate genes associated with adaptation to salinity change in *Triplophysa* loaches. Genes marked in red were associated with adaptation to salinity changes in both *T. stewarti* and *T. stenura*. Genes marked in black were identified in *T. stewarti*. The schematic model in which genes are assigned to different functional categories was adapted from Nguyen et al. (2016). *KCNG4*: potassium voltage-gated channel subfamily G member 4, *ABCC5*: C member 5 multi-specific organic anion transporter 5, *DPP6*: dipeptidyl aminopeptidase-like protein 6, *IL1RAPL1A*: interleukin-1 receptor accessory protein-like 1-A, *GLUT1*: facilitated glucose transporter member 1, *HNF4A*: hepatocyte nuclear factor 4-alpha, *PPARA*: peroxisome proliferator-activated receptor alpha, *GATA2*: GATA-binding factor 2, *GGH*: gamma-glutamyl hydrolase, *NLRC5*: NOD-like receptor C5, *PRDX1*: peroxiredoxin-1, *CDH13*: cadherin-13, *CADM1*: cell adhesion molecule 1, *PTPDC1*: protein tyrosine phosphatase domain-containing protein 1, *XIRP2*: Xin actin-binding repeat-containing protein 2, *HYDIN*: hydrocephalus-inducing protein-like protein, *APC*: adenomatous polyposis coli protein, *MAP2K1*: dual specificity mitogen-activated protein kinase 1.

A catalog of genes involved in ion transport, energy metabolism, structural reorganization, immune response, detoxification, and signal transduction may be responsible for adaptation to salinity change in *Triplophysa* loaches (Figure 4). A total of 26 genes were commonly identified with both unsupervised and supervised approaches to be associated with adaptation to salinity change in *T. stewarti*, and they are involved in energy metabolism to maintain osmotic pressure, ion transport, structural reorganization, and signal transduction to transduce signals in osmosensing (Supplementary Table S4). Of genes involved in energy metabolism, *HNF4A* is involved in the transcriptional regulation of long-chain polyunsaturated fatty acid biosynthesis (Dong et al., 2016), and it is assumed to play an important regulatory role in osmotic acclimation in rabbitfish (Wang et al., 2018), killifish (Whitehead et al., 2012), and striped catfish (Schmitz et al., 2017). *GLUT1* is responsible for glucose uptake and energy supplementation under salinity stress (Mueckler, 1994), and earlier studies showed that the expression of *GLUT1* is upregulated in response to salinity increase in sea bream (Balmaceda-Aguilera et al., 2012) and striped catfish (Nguyen et al., 2016). Of genes related to ion transport, *DPP6* promotes cell surface expression and modulates the activity and gating characteristics of the potassium channel KCND2 (Soh and Goldstein, 2008; Seikel and Trimmer, 2009). Of 39 genes identified to be associated with adaptation to salinity changes in both *T. stewarti* and *T. stenura* using unsupervised genetic differentiation methods, *KCNG4* is presumed to be involved in ion transport under osmotic stress (Mederos et al., 2009), and genes (e.g., *CDH13*, *CADM1*, and *PTPDC1*) are involved in structural reorganization during cell volume change under salinity stress (Nguyen et al., 2016). Genes, *CBFB* and *NLRC5*, are identified to be associated with salinity change with BAYENV2 in both *Triplophysa* species. *NLRC5* was involved in the immune response during the smoltification process in Atlantic salmon (Pontigo et al., 2016), marine-freshwater divergence in three-spined stickleback (Terekhanova et al., 2014), and biological invasions in round goby (Adrian-Kalchhauser et al., 2020). Of 20 common DEGs identified in both *Triplophysa* species, the *APC* gene may function in Wnt signaling by promoting the rapid degradation of CTNNB1 (Aoki and Taketo, 2007), and Wnt signaling has been demonstrated to involve a marine-freshwater divergence in three-spined sticklebacks (Yu et al., 2009; Jones et al., 2012). Interestingly, *WNT7B* involved in Wnt signaling is associated with adaptation to salinity change in *T. stewarti* (Supplementary Table S4).

### Methodological consideration

Graph learning has rarely been used in population genomics data analysis (but see McKinney et al., 2020). However, the graph learning-based method should be applied to identify groups of loci that showed similar evolutionary signals. Our newly developed LD graph learning method produced consistent results with the previously developed three-step LDna method (Fang et al., 2020). The large proportion of overlapping highly differentiated SNPs between our LD graph learning and three-step LDna (Supplementary Figure S3) and disproportionally more SNPs identified as salinity-associated using EAA also identified as highly differentiated in LD graph learning and three-step LDna validate the effectiveness of our newly developed LD graph learning method. Instead of calculating LD values measured by *r* ^2^ statistic between every two pairwise SNPs or between SNPs within a limited distance (e.g., a distance of 100 SNPs in three-step LDna), we modeled the correlation between one SNP and all the other SNPs simultaneously and used these correlation coefficient values to represent the correlation between SNPs. In addition, our LD graph learning method incorporates only two steps and uses fewer parameters (e.g., no need to set *λ*_*lim*_ and |*E*|_*min*_ in LDna and the derived three-step LDna). According to the LDna method (Kemppainen et al., 2015), the values of *λ*_*lim*_ and |*E*|_*min*_ will affect the clustering of SNPs, though LD clusters are robust to these parameter settings. Compared with early developed LDna (Kemppainen et al., 2015) and network analysis of linkage disequilibrium (McKinney et al., 2020), our LD graph learning method can handle up to millions of loci, which is memory-saving and more applicable to re-sequencing data. Our script also supports parallel computing.

In conclusion, by integrating population genomic, transcriptomic, and electron probe microanalysis approaches, we unraveled the population differentiation patterns and possible adaptive mechanisms to salinity change in two *Triplophysa* species: *T. stewarti* and *T. stenura*. Our results showed contrasting population differentiation patterns between the two species, which may be attributed to their different ecological characteristics and population histories. Using both unsupervised and supervised genetic differentiation methods, including our brand-new LD graph learning method, we found that the two *Triplophysa* species showed limited genetic signals of parallel adaption to salinity changes from either genetic or gene expression variation perspective. However, a catalog of genes involved in iron transport, energy metabolism, structural reorganization, immune response, and signal transduction may function in adaptation to salinity change in *Triplophysa*, of which several genes are also identified in other fishes. These findings help predict how aquatic organisms will respond to similar selective pressures on the QTP when facing challenges from climate changes.

## Data Availability

SNPs and quantitative expression data based on RNA-seq underlying this study have been deposited in Dryad https://doi.org/10.5061/dryad.x3ffbg7mr. Code for the newly developed unsupervised linkage disequilibrium graph learning method has been deposited in Zenodo https://doi.org/10.5281/zenodo.6422021.
