# Supplementary material for: Contrasting population differentiation in two sympatric Triplophysa loaches on the Qinghai–Tibet Plateau
Source: Front Genet. 2022 Aug 25;13:958076. doi: 10.3389/fgene.2022.958076 (PMC9452750; doi:10.3389/fgene.2022.958076)
Supplement: Supplementary file 1 [file DataSheet1.docx]

Supplementary Material

# Supplementary Figures

**Figure legends**

**Supplementary Figure S1** PCA from ‘common’ SNP dataset (A) and ‘individual’ SNP datasets in *T. stewarti* (B) and *T. stenura* (C).

**Supplementary Figure S2** Population stratification in the two species, *T. stewarti* (A) and *T. stenura* (B), using ADXIMTURE analyses. The optimal K is 3 and 1, respectively.

**Supplementary Figure S3** Number of SNPs identified using unsupervised and supervised methods in *T. stewarti*. (A) Genome-wide SNP cluster 2 of four *T. stewarti* populations identified using LD graph learning. (B) Highly differentiated chromosome-wide SNP clusters within genome-wide SNP cluster 2 in (A). (C) Overlap of SNPs in highly differentiated genome-wide SNP clusters among high- and mid-salinity *T. stewarti* population pairs. (D) LD-cluster 2 identified using three-step LDna. (E) SNPs associated with salinity identified using BAYENV2.

**Supplementary Figure S4** (A) The 11 LD-clusters in *T. stewarti* identified by three-step LDna method on the single linkage clustering tree of the final step. (B) PCA of the 11 LD-clusters. Triangles and circles indicate high- and mid-salinity populations. (C, D) Comparisons of fixation index *F*_ST_ (C) and absolute population differentiation *D*_xy_ (D) between SNPs in LD-cluster 2 (green dots) and all SNPs (gray dots). Gray dash line indicates the average of *F*_ST_ and *D*_xy_ calculated using all SNPs.

**Supplementary Figure S5** (A) The 16 SOCs in *T. stenura* identified by three-step LDna method on the single linkage clustering tree of the final step. (B) PCA of the 16 SOCs. Triangles and circles indicate mid- and low-salinity populations.


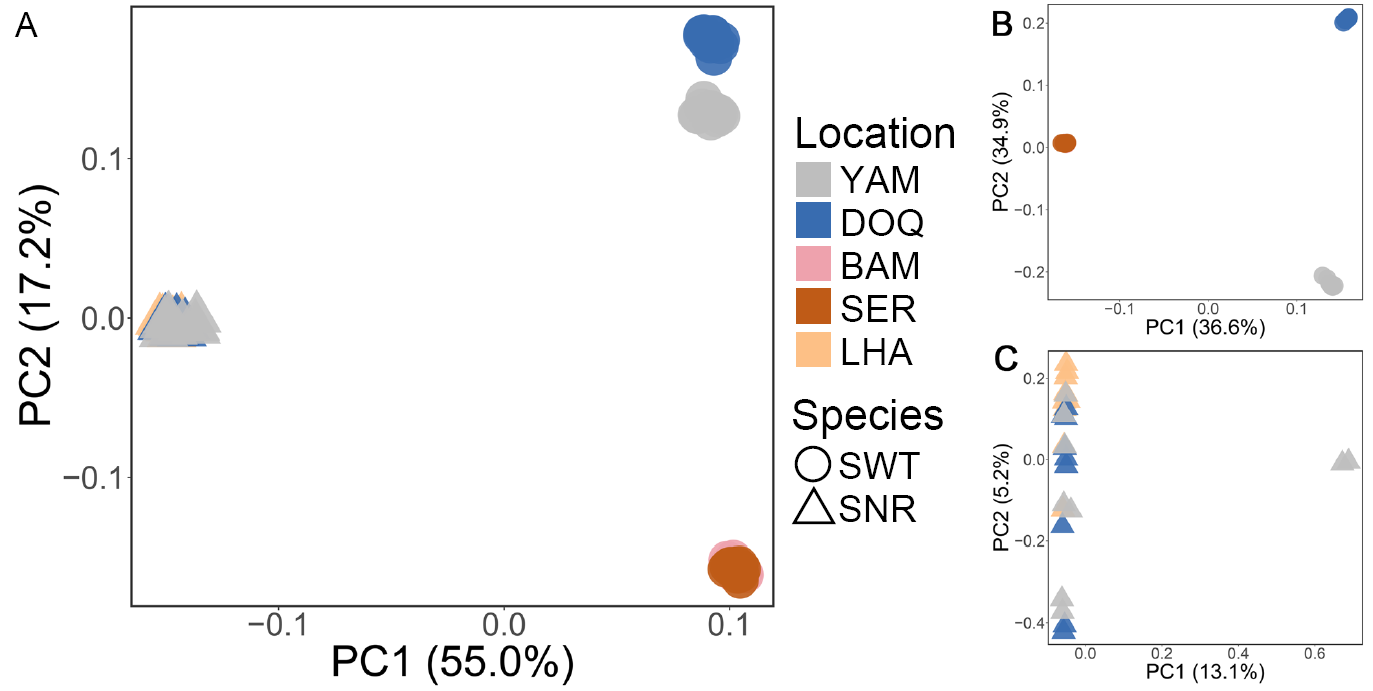


**Supplementary Figure S1**


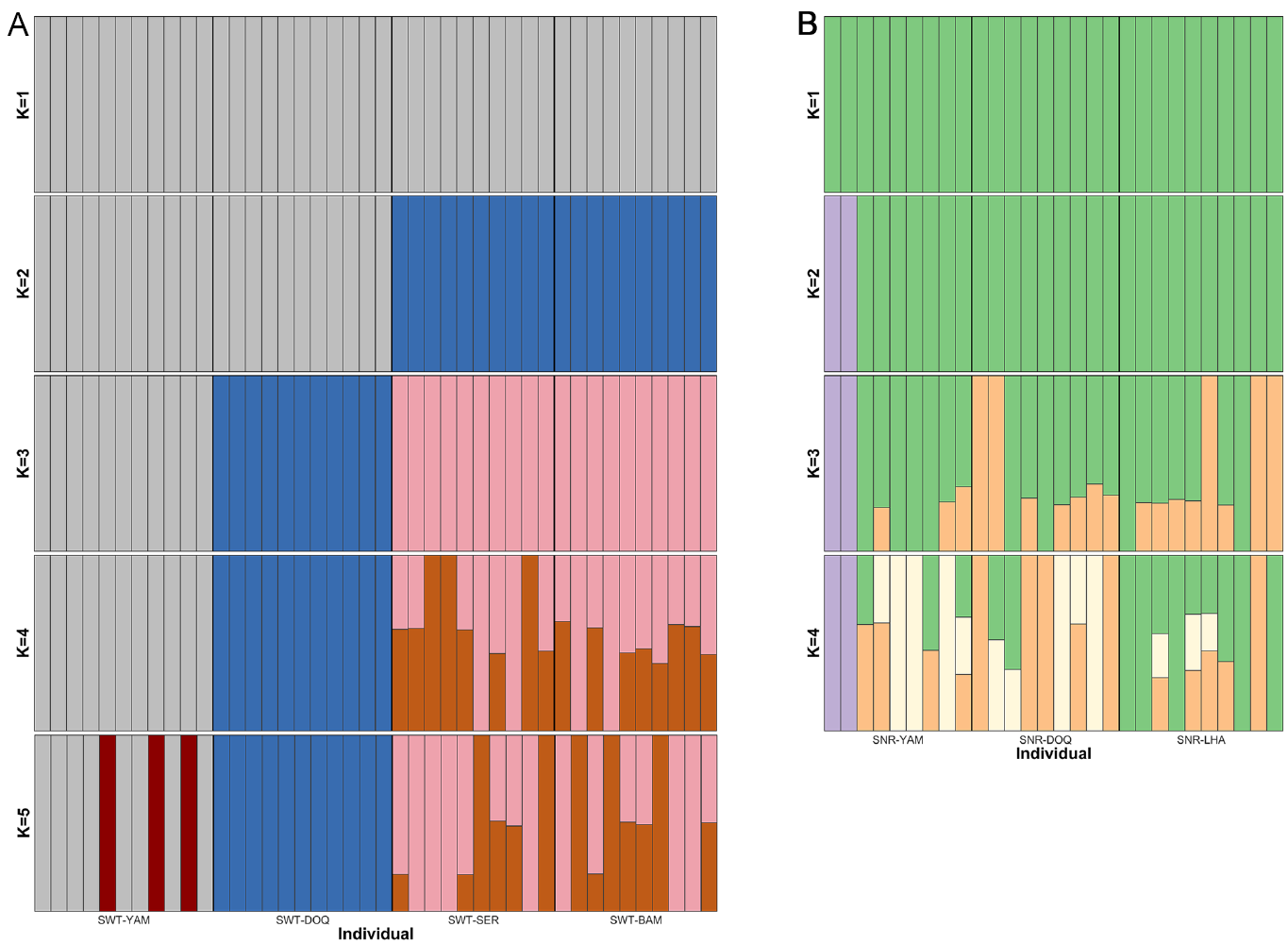


**Supplementary Figure S2**


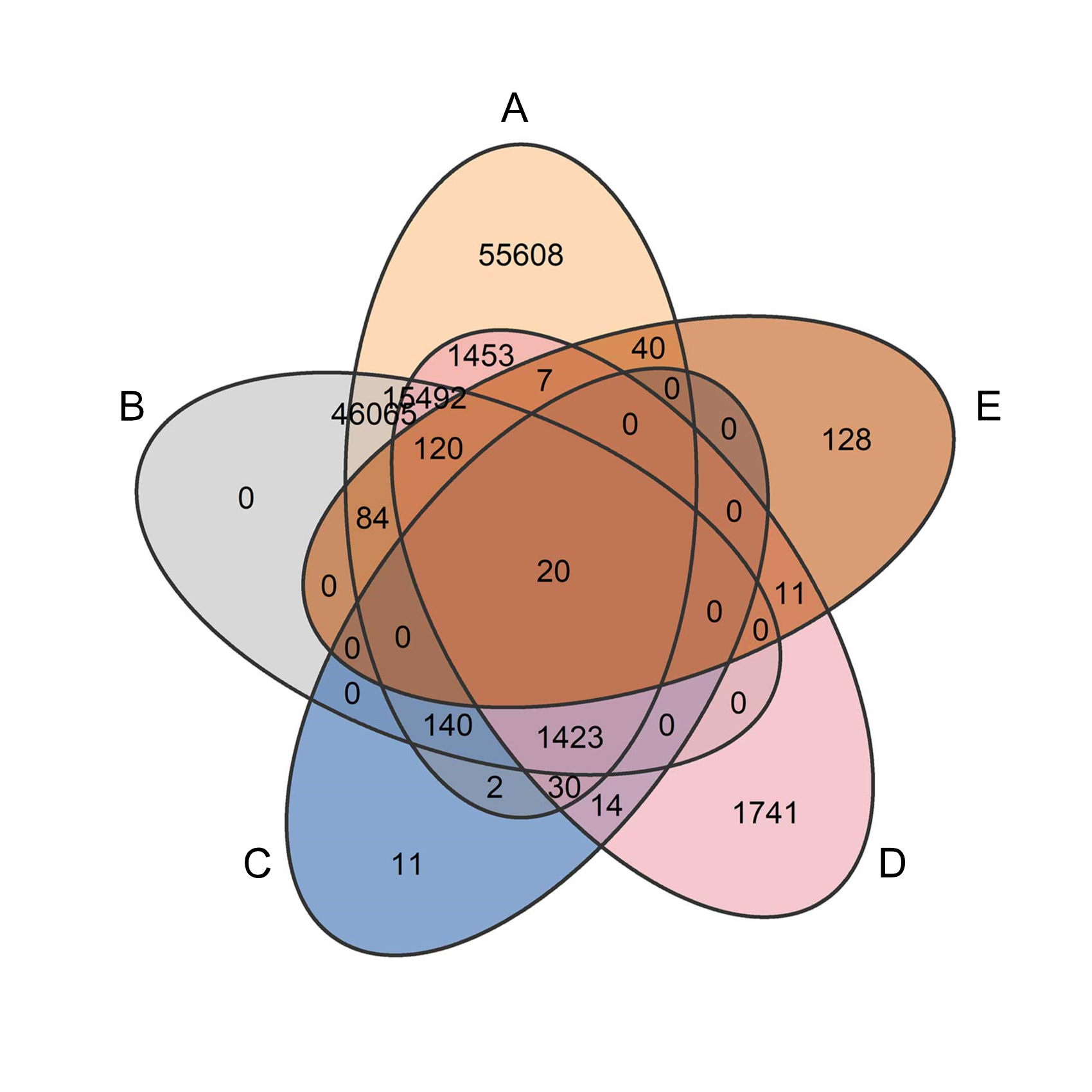


**Supplementary Figure S3**


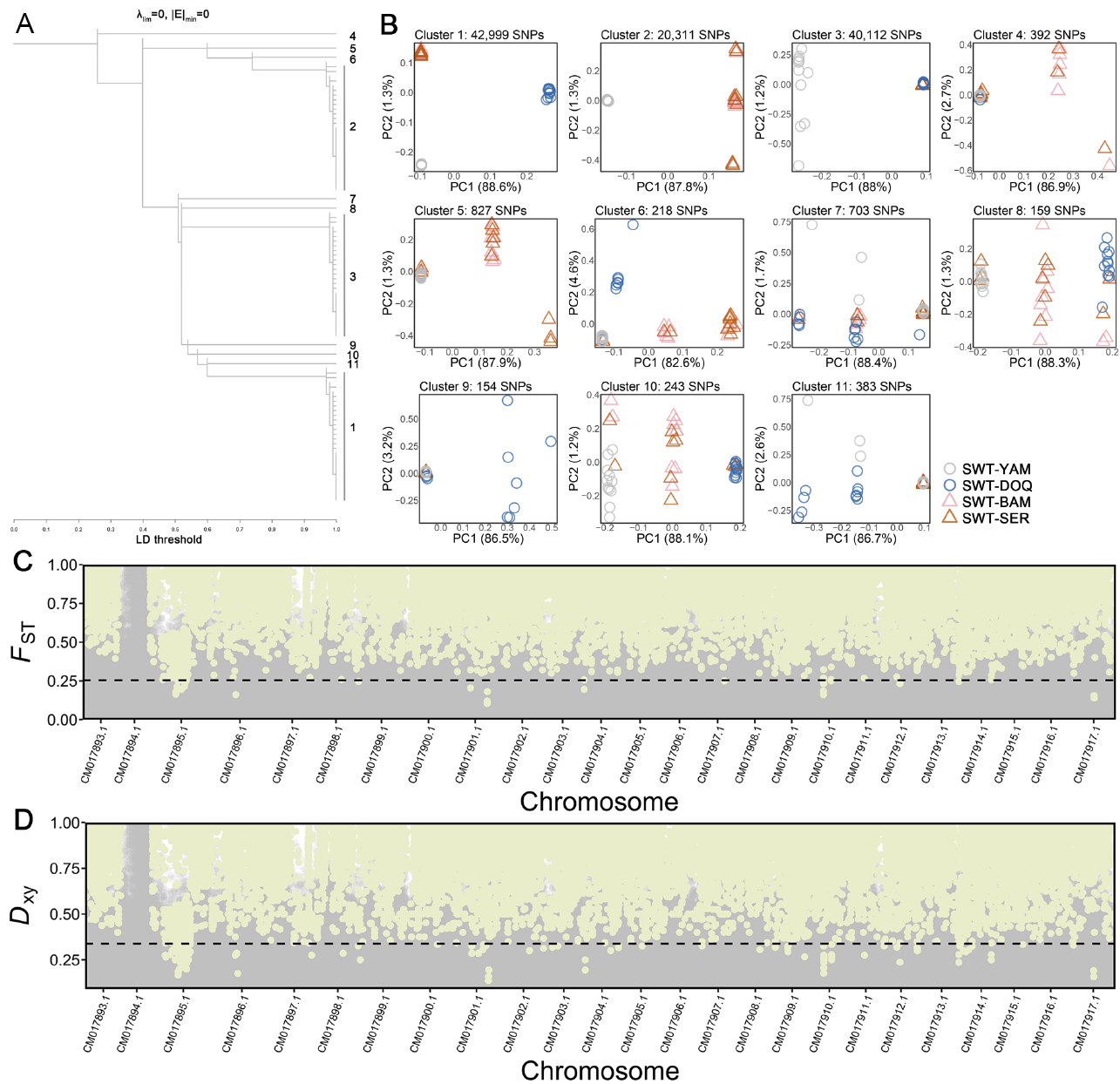


**Supplementary Figure S4**


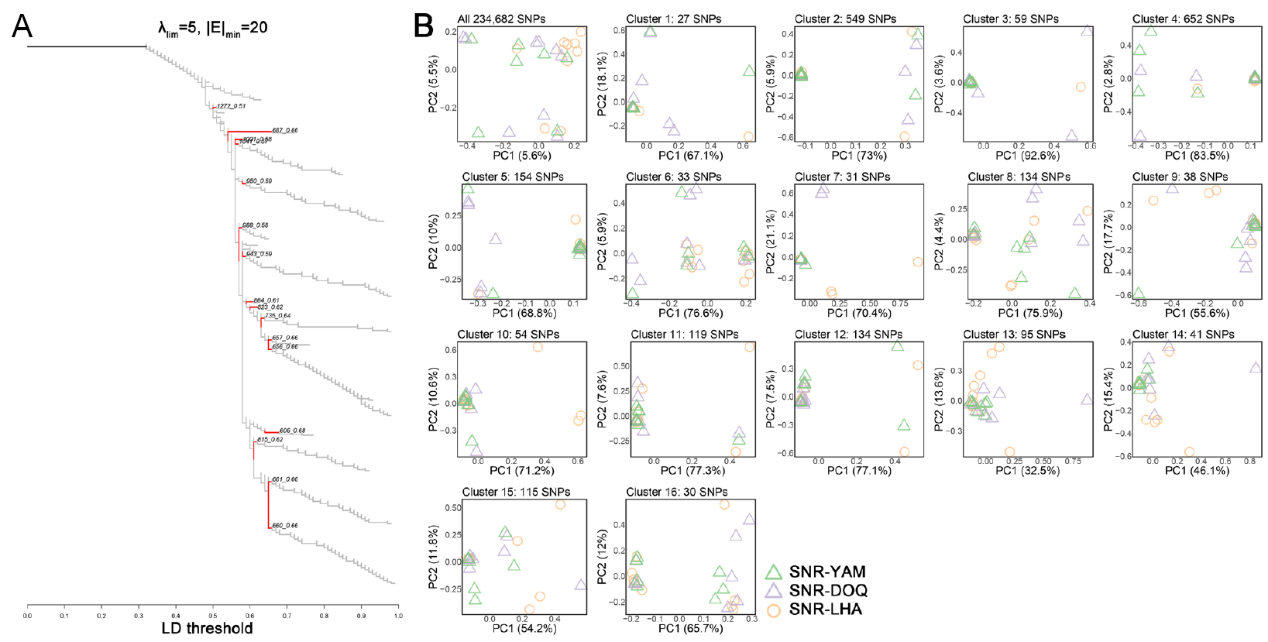


**Supplementary Figure S5**
